# Effect of Antioxidant Water on the Bioactivities of Cells

**DOI:** 10.1155/2017/1917239

**Published:** 2017-08-17

**Authors:** Seong Gu Hwang, Ho-Sung Lee, Byung-Cheon Lee, GunWoong Bahng

**Affiliations:** ^1^Department of Animal Life and Environmental Science, Hankyong National University, Anseong 17579, Republic of Korea; ^2^Institute for Information Technology Convergence, Division of Electrical Engineering, Korea Advanced Institute of Science and Technology, Daejeon 34138, Republic of Korea; ^3^Department of Mechanical Engineering, The State University of New York Korea, Incheon 21985, Republic of Korea

## Abstract

It has been reported that water at the interface of a hydrophilic thin film forms an exclusion zone, which has a higher density than ordinary water. A similar phenomenon was observed for a hydrated hydrophilic ceramic powder, and water turns into a three-dimensional cell-like structure composed of high density water and low density water. This structured water appears to have a stimulative effect on plant growth. This report outlines our study of antioxidant properties of this structured water and its effect on cell bioactivities. Culturing media which were prepared utilizing this antioxidant structured water promoted the viability of RAW 264.7 macrophage cells by up to three times. The same tendency was observed for other cells including IEC-6, C2C12, and 3T3-L1. Also, the cytokine expression of the splenocytes taken from a mouse spleen increased in the same manner. The water also appears to suppress the viability of cancer cell, MCF-7. These results strongly suggest that the structured water helps the activities of normal cells while suppressing those of malignant cells.

## 1. Introduction

There have been many arguments on the role of water in a cell and this debate still continues today [1]. Most of these debates are still primitive, focused on whether water is a simple solvent medium of diverse organic components in cytoplasm or it plays an essential role [2]. As the name suggests, most studies in molecular biology have been conducted focusing on the behavior of molecular components such as lipids, proteins, and other cellular molecules including various antioxidants. Despite the fact that water occupies the largest portion of a cell, it does not receive proper attention from academia in biology and medicine, likely because the structure of water and its effect on biological activities is still not yet well understood [3–5].

Recently it was claimed that there is a fourth phase of water similar to a liquid crystal, in addition to the three phases of water: solid, liquid, and gas [6]. This fourth phase takes place at the interface of a hydrophilic material such as Nafion® film as water molecules are arranged along the polarity of the surface. Since its density is higher than ordinary water, microspheres in a suspension are excluded as the water is structured, and, based on this phenomenon, it was named as an exclusion zone [7–9]. Also, electric potential as high as −200 mV has been observed to develop across the boundary of the exclusion zone and outside of this region (exclusion zone negative). This potential is generated by the dissociation of water molecules into negative ions (OH-) and protons as it is structured [6]. This important finding implies that water itself can affect the growth and bioactivity of live beings.

Actually, it was reported that an exclusion zone is formed also around hydrophilic ceramic powder and can stimulate seed germination and early sapling growth in brown chickpea seeds. Root length and/or formation of shoots increased at least 2~3-fold [10]. In due course, structured water may also influence cell bioactivities. To confirm this possibility, an evaluation of the property of structured water composed of an exclusion zone and its effect on the bioactivities of cells are necessary. In this study, structured water formed by mixing with a hydrophilic ceramic powder was discovered to have an antioxidant property. In addition, to address any arguments regarding the effects of any possible dissolved mineral components, we have developed another method utilizing far-infrared ray waves from the ceramic powder to make structured water in a noncontact manner.

This structured water was utilized to prepare culture media for evaluating its effect on the bioactivities of cells, and this water appears to have a meaningful effect on the bioactivities of normal cells such as cell viability, phagocytic activity, and natural killer cell activity. Conversely, the viability of cancer cell was suppressed when it was incubated with the same medium made of structured water. These results imply that the role of water should be understood as a critical component rather than merely as a simple media in a cell.

## 2. Materials and Methods

### 2.1. Evaluation of Antioxidant Property of Water

#### 2.1.1. Water Mixed with the Ceramic Powder

A hydrophilic ceramic powder, QELBY®, with particle size ranging from 40 nm to 1 *μ*m, was obtained from Quantum Energy Co., Ltd. It was produced by finely grinding natural clay minerals of the feldspar family with silica as the main component (more than 60%).

**Figure 1:**
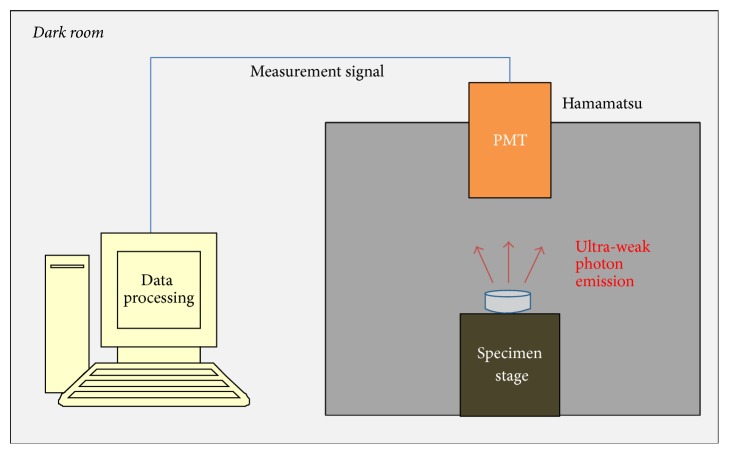
Schematic diagram of the ultra-weak photon emission measurement system.

To evaluate the antioxidant property, an ultra-weak photon emission was measured using a photomultiplier tube (Hamamatsu Photonics, E1341-01) under total darkness.  Figure 1 shows the schematic diagram of the photon emission measurement system.

For the evaluation of the antioxidant property of water mixed with the powder, three kinds of samples were prepared. The same amount of bean sprout stems were mixed with deionized water only; deionized water mixed with natural silica powder; and deionized water mixed with QELBY powder, respectively. The concentration of the powder in each mixture was 0.01 g/ml. The number of photon emissions per one second was measured for 8 minutes and an average value was calculated. The measurement was repeated every day for seven days. In addition, the color change of the bean sprout stems was observed simultaneously.

#### 2.1.2. Water Treated in the Noncontact Method

**Figure 2:**
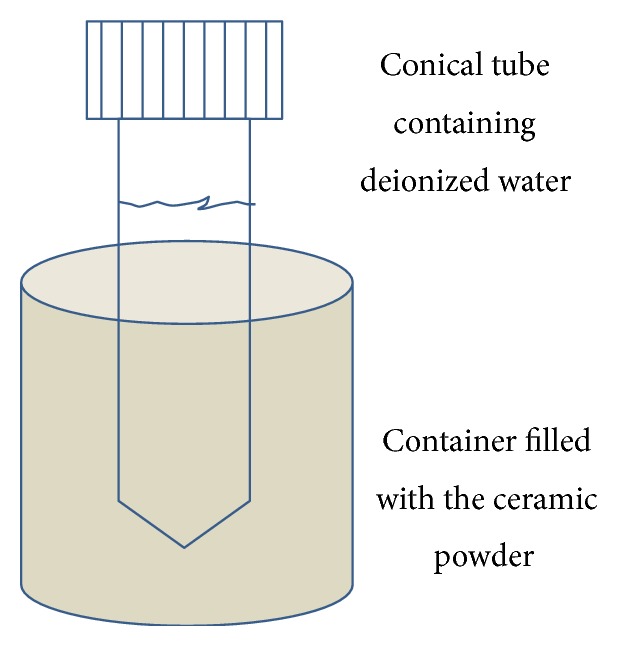
*Preparation of water in the noncontact method*. Deionized water in a 50 ml vial was put up into the ceramic powder for two days and used for the preparation of culturing media by mixing with the powder form DMEM.

For the noncontact treatment of water, vials containing 50 ml of deionized water were put up into the ceramic powder halfway for 48 hours before it was used for the preparation of the culturing media. Deionized water was obtained by a water purification system that produced an ultra-pure water type I grade with electrical resistivity of 18.2 MΩcm and reduction of inorganics up to 99.99% (Young Lin Instrument, aqua MAX™ ultra 370 series).  Figure 2 shows the schematic diagram of water treatment in a noncontact manner.

To evaluate the antioxidant property of water prepared in the noncontact method, the number of photons emitted per second was measured every day for 3 minutes at a time after adding 0.5 ml of 10 mM tert-Butyl hydroperoxide (TBHP) to 1 ml of deionized water (control) and noncontact treated deionized water (sample), respectively, for the duration of 4 days. TBHP, as an organic peroxide, is a strong oxidant and forms radical species of the form RO^*∗*^ during the decomposition of its O-O bond [11]. It is more stable to compare with H_2_O_2_ and widely used as a source of free radical in cellular activity as well as an inducer of oxidative stress [12, 13].

### 2.2. Preparation of Culture Media

#### 2.2.1. Culture Media Prepared by Mixing with Ceramic Powder

To avoid any possible contamination, the QELBY ceramic powder was heat treated using an electric muffle furnace for an hour at 800°C and then autoclaved just before mixing with Dulbecco's modified Eagle's medium (DMEM). DMEM with 10% of heat inactivated fetal bovine serum (FBS) as a supplementation and 1% penicillin-streptomycin (P/S) was prepared by mixing the powder form DMEM (Gibco®, 12800-017, USA) with deionized water. The liquid form DMEM was mixed with 1% in weight concentration of the powder and agitated for 5 minutes and then left for 2 days in a refrigerator maintained at 4°C. After 2 days, the suspended layer was filtered with Whatman® filter paper (grade 5) and the weight of the filtered paper was measured in order to estimate the total dissolved powder in the media. The filtered DMEM was diluted with a new fresh DMEM to certain concentrations (e.g., 25, 50, 100, 200, and 400 *μ*g/ml) prior to preparing the culturing media.

The concentration of the powder in the filtered DMEM was calculated to be about 700 ppm~750 ppm. The final concentration of powder in the diluted media was estimated to be lower than 1 ppm. For the natural killer cell and splenocyte cell, RPMI 1640 medium was used instead of DMEM. Supplementary drawings SFig. 1~3, in Supplementary Material available online at https://doi.org/10.1155/2017/1917239, show the overall diagram of media preparation and cell viability measurement.

#### 2.2.2. Culture Media with the Water Prepared in the Noncontact Method

Another set of culture media was prepared using a structured water prepared in a noncontact manner. A centrifuge conical tube containing 50 ml of deionized water was inserted into the ceramic powder for 48 hours or other specified durations at room temperature. Deionized water treated in this way was used to prepare the culture media by mixing with the DMEM powder. Supplementary drawing SFig. 4 shows the overall diagram of media preparation and cell viability measurement for the noncontact method.

### 2.3. Evaluation of Bioactivities of Cells

#### 2.3.1. Viability of Macrophage Cell

Murine RAW 264.7 was obtained from the Korean Cell Line Bank (KCLB, Korea). To evaluate the viability of the macrophage cell, it was seeded in a 96-well plate at a density of 1 × 10^5^ cells/ml in a final volume of 100 *μ*l and allowed to adhere for 3 hours. It was then pretreated for 1 hour with 10 *μ*g/ml lipopolysaccharide (LPS) for the stimulation before applying the prepared media. After 1 hour, prepared media with increasing concentrations of dissolved QELBY ceramic powder was added to each well before incubation. Incubation was performed for 24 hours or 48 hours at 37°C in humidified 5% CO_2_ atmosphere. After the incubation, media in the wells were suctioned and 100 *μ*l of fresh media with 10 *μ*l of CCK-8 solution were added per well and then incubated at 37°C for 2 hours. A cell counting kit (CCK-8, Dojindo, Japan) was used after 2 hours of treatment for the determination of cell viability using enzyme-linked immunosorbent assay (ELISA) microplate reader (TECAN, Switzerland) for absorbance at 450 nm. As a control, the viability of the cell cultured in DMEM without LPS stimulation was measured. The viability of the cell with LPS stimulation was measured also as a positive control. The viability of the treated cells was expressed as the percentage of control cells. A schematic diagram of the media preparation and cell viability evaluation is described in the supplementary drawing SFig. 1.

#### 2.3.2. Phagocytic Activity

The phagocytic activity of a macrophage cell was evaluated by measuring the uptake of neutral red dye. Cells were cultured at 37°C with the prepared media in humidified 5% CO_2_ for 24 hours and then 100 *μ*l of 0.075% aseptic neutral red solution was added and cultured again for 1 hour. Following the end of culture, the plate was washed three times with phosphate-buffered saline, and a mixture of 100% ethanol and 99.9% acetic acid (1 : 1 v/v) was added to the 150 *μ*l of cell lysate. The mixture was mixed fully and absorbance at 550 nm was measured using an ELISA reader. The schematic diagram of the media preparation and evaluation of the phagocytic activity is shown in the supplementary drawing SFig. 2.

#### 2.3.3. Natural Killer Cell Activity

For the evaluation of the natural killer (NK) cell activity and viability of splenocyte, mice (C57BL/6) were sacrificed and their spleens were collected aseptically. To prepare the media, 0.1 g of QELBY ceramic powder was mixed with 10 ml of Roswell Park Memorial Institute (RPMI) 1640 medium containing 10% fetal bovine serum (FBS) and 1% penicillin-streptomycin and stood for 24 hours at 4°C. The suspended layer in differing amounts were used for the preparation of culture media, for example, 25, 50, 100, and 200 *μ*g/ml by mixing with a new fresh RPMI 1640 medium.

Target cell (YAC-1) was seeded in a 96-well plate at a density of 5 × 10^3^ cells/ml for 3 hours. An effector cell (isolated splenocyte) and target cells were mixed at effector : target ratio of 10 : 1 and incubated with the prepared media for 24 hours. NK cell activity was assessed after treatment with CCK-8 assay solution for 2 hours. The NK cell activity was calculated as follows based on the optical density (OD) measured by ELISA reader at 450 nm.

(1) % NK cell activity=1−ODeffector+target−ODeffectorODtarget×100.

Supplementary drawing SFig. 3 shows the overall cell culture process and natural killer cell activity measurement.

#### 2.3.4. Splenocyte and Cytokine Expression

Mice spleens were collected aseptically to prepare spleen cell suspension. The evaluation of splenocyte viability was carried out according to the CCK-8 method. Splenocytes were seeded in a 96-well plate at 1 × 10^5^ cells/ml for 1 hour. The prepared media with different amounts of the suspended layer, 0, 25, 50, 100, and 200 *μ*g/ml, from the RPMI 1640 mixed with the 1% of QELBY powder stood for 48 hours, were added to the cells and incubated for 24 and 48 hours. CCK-8 reagent was added to the cell suspension and the optical density was measured 2 hours later at 450 nm using a microplate reader.

Changes in cytokine expression of murine splenocytes were measured also. Splenocytes were seeded in a 6-well plate at 5 × 10^5^ cells/ml for 3 hours and then incubated for 24 hours with the prepared culture media. Total RNA was extracted using TRIzol® reagent (Takara Korea, Korea) according to the manufacturer's instructions. One *μ*g of RNA was used to acquire the complementary DNA (cDNA) using the protocol provided by M-MuLV reverse transcriptase (Fermentas, Lithuania) for reverse transcription polymerase chain reaction (RT-PCR). Specific primers were used to amplify different genes. PCR products were then separated by electrophoresis using 1.5% agarose stained with ethidium bromide, followed by a UV transillumination. A PCR analysis was done three times and a densitometry analysis was carried out using Lane® 1D software. Beta actin (*β*-actin), interleukin-12 (IL-12), and interferon-gamma (INF-*γ*) expressions were analyzed.

#### 2.3.5. Breast Cancer Cell

The viability of the human breast cancer cell, MCF-7, was measured in the same way using both kinds of culture media. The suspended layer was taken in different amounts from the premixed DMEM with 1% of QELBY ceramic powder after 48 hours of storage in a refrigerator maintained at 4°C. Additionally, to see the effect of water preparation process, DMEM mixed with the 1% of QELBY powder was agitated for 3 minutes and then centrifuged at 3,000 rpm for 5 minutes. Different amounts of the supernatant were collected and used for the preparation of culture media.

Also, the DMEM culture medium which was prepared using only the water that was treated for 48 hours in a noncontact method was used for the evaluation of cell viability.

### 2.4. Statistical Analysis

All experiments were performed for the specified number of *n* indicated in the figure captions, from *n* = 4 to *n* = 7, and results are presented as mean ± SD. The cellular activities in intact cultures were taken for 100% of the cultures. Statistical significance was determined using a one-way analysis of variance (ANOVA) followed by Duncan's Multiple Range Test. A value of *p* < 0.05 was considered statistically significant.

## 3. Results

### 3.1. Antioxidant Property of Water

#### 3.1.1. Water Mixed with the Powder

**Figure 3:**
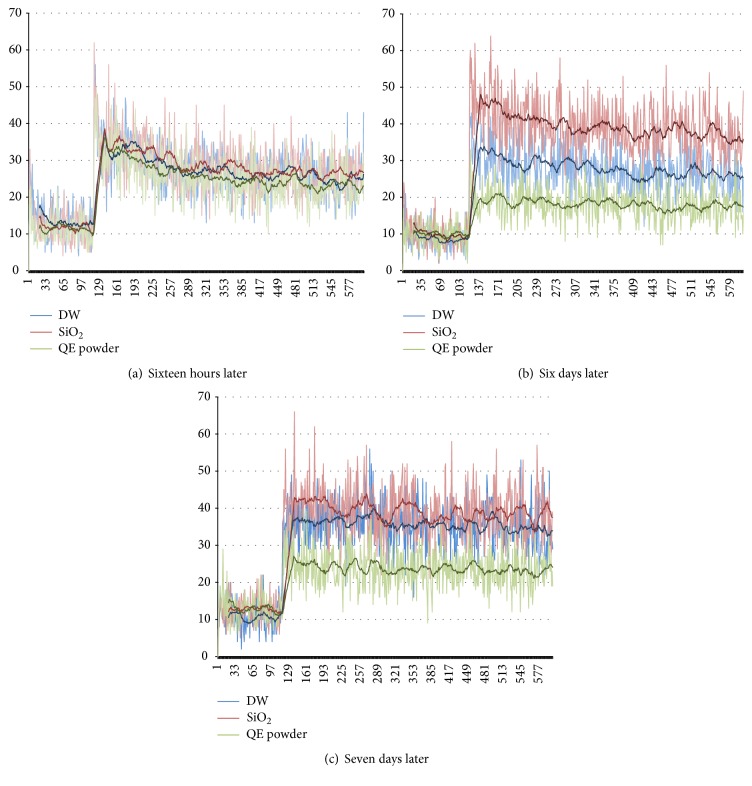
*Measurement of ultra-weak photon emission with a photomultiplier tube*. The blue line (DW), red line (SiO_2_), and the green line (QE powder) indicate the number of photons from deionized water only, deionized water mixed with silica powder, and deionized water mixed with the QELBY powder, respectively. The number of photons per one second was measured for eight minutes. The vertical axis depicts the number of photons, and the horizontal axis represents seconds elapsed.

The QELBY® powder showed a good hydrophilic property and colloid formation was observed when mixed with water. For the bean sprout stems mixed with water prepared in a contact method, the numbers of photons emitted are not much different among the three specimens measured 16 hours later as shown in Figure 3(a). However, the number of photon emissions from the deionized water mixed with silica powder (red line) increased after 6 days followed by the deionized water only (blue line) as shown in Figure 3(b). After seven days, it was almost the same as shown in Figure 3(c). In comparison, the deionized water mixed with the QELBY® ceramic powder (green line) clearly showed a lower photon emission even after seven days as shown in Figures 3(b) and 3(c).  Table 1 shows the average number of ultra-weak photon emission per one second measured for eight minutes from the bean sprout stems mixed with different kinds of water prepared in the contact method.

**Figure 4:**
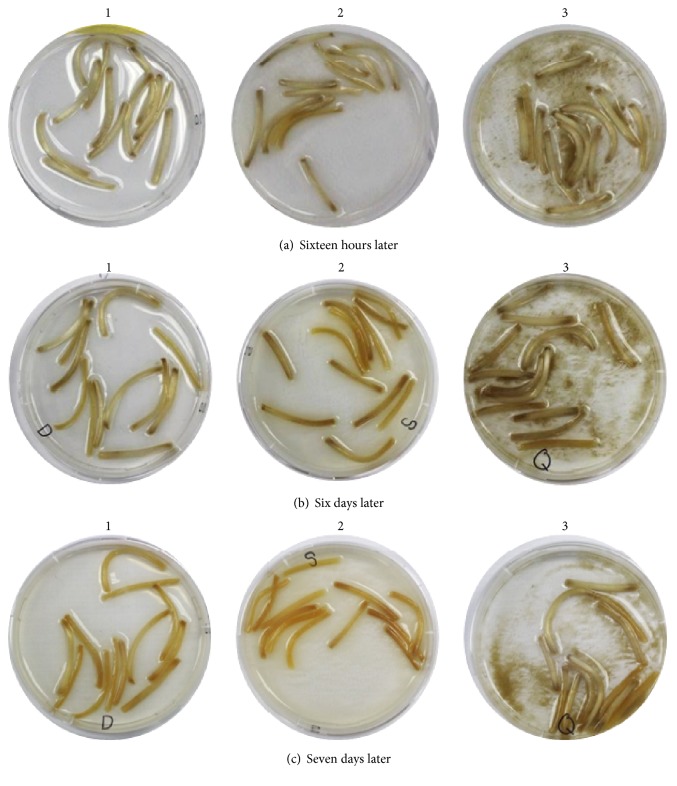
*Observation of the color change of bean sprout stems.* (1) Deionized water; (2) deionized water mixed with silica powder; (3) deionized water mixed with QELBY ceramic powder, respectively.

In the deionized water or deionized water mixed with the silica powder as shown in Figures 4(a), 4(b), and 4(c), the color of the bean sprout stems turned dark brown or brown. However, the color of the bean sprout stems stored in the deionized water mixed with the QELBY ceramic powder did not change significantly and was light brown after seven days as shown in Figure 4(c). These results indicate that bean sprout stems in the deionized water and the deionized water mixed with silica powder oxidized faster than those stored in the deionized water mixed with the QELBY powder.

**Figure 5:**
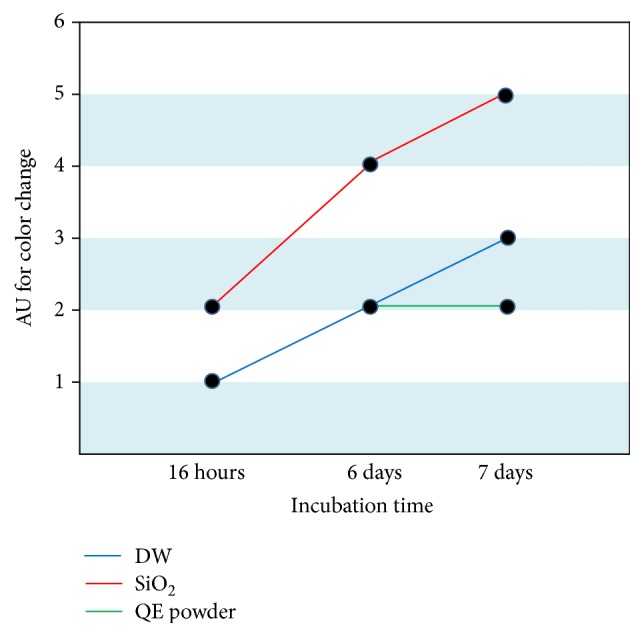
*Variation of color of the bean sprout stems with incubation time*. Arbitrary unit (AU) in 5-point scale is adopted to indicate the color change, that is, from AU1, white, and no change in color, to AU5, dark brown, and severely changed.

In order to indicate the change of color quantitatively, an arbitrary unit from 1 (white, no change) to 5 (dark brown, severely changed) has been adopted.  Figure 5 shows the variation of color with the incubation time, 16 hours, 6 days, and 7 days, for the bean sprouts shown in Figure 4. Until 6 days of incubation, there was no difference in color between deionized water only and in deionized water mixed with QELBY ceramic powder. Next day, on the 7th day of incubation, the color of the bean sprouts in the deionized water mixed with QELBY® powder hardly changed but that of the bean sprouts in deionized water began to change. The color of bean sprouts in deionized water mixed with silica powder rapidly changed continuously from white to dark brown from the beginning all the way to the last 7th day.

#### 3.1.2. Water Treated in the Noncontact Method

**Figure 6:**
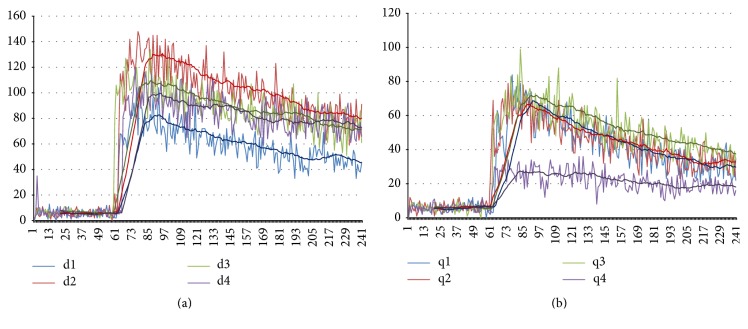
*Results of ultra-weak photon emission measurements after mixing 0.5 ml of 10 mM tert-Butyl hydroperoxide with 1 ml of deionized water*. The duration of measurement is 3 min. The vertical axis represents the number of photons per second and the horizontal axis represents seconds elapsed. (a) Deionized water. The number of photon emissions increased compared to day 1 (d1, d2, d3, and d4 indicate the day of measurement, resp.). (b) Deionized water treated in the noncontact manner. The number of photon emissions decreased drastically at day 4 (q1, q2, q3, and q4 indicate the day of measurement, resp.).

Experimental measurements show that the photon emission from the deionized water increased on the second day of measurement. On days 3 and 4, the number of photon emissions was lower than those from day 2. However, they were still higher than that of day 1 as shown in Figure 6(a). In comparison, the number of photon emissions from the deionized water treated in a noncontact manner showed a dramatic decrease on day 4, reaching one-fourth of its initial level after 93 h of exposure, as shown in Figure 6(b).

**Figure 7:**
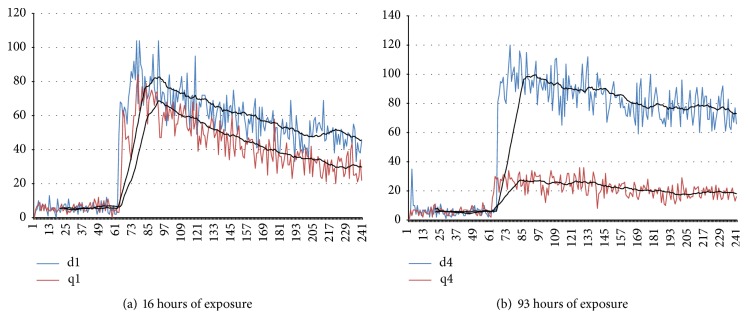
*Results of the ultra-weak photon emission measurement of deionized water (blue line) and noncontact treated deionized water (red line), respectively*. (a) 16 hours later. (b) 93 hours later. The antioxidant property levels of the noncontact treated water is about one-fourth of the control.

Figure 7 is redrawn from the data shown in Figure 6 for direct comparison to the control data. After 16 hours of the noncontact treatment, the treated water already shows a lower number of photon emissions compared to the control as shown in Figure 7(a). This difference increased after 93 hours of exposure and its level was about one-fourth of the control (Figure 7(b)). This indicates that the antioxidant property of the water treated in the noncontact manner increased as exposure time increased.

### 3.2. Effect of Culturing Media on the Cell Viability

#### 3.2.1. Culturing Media Prepared by Mixing with Powder

**Figure 8:**
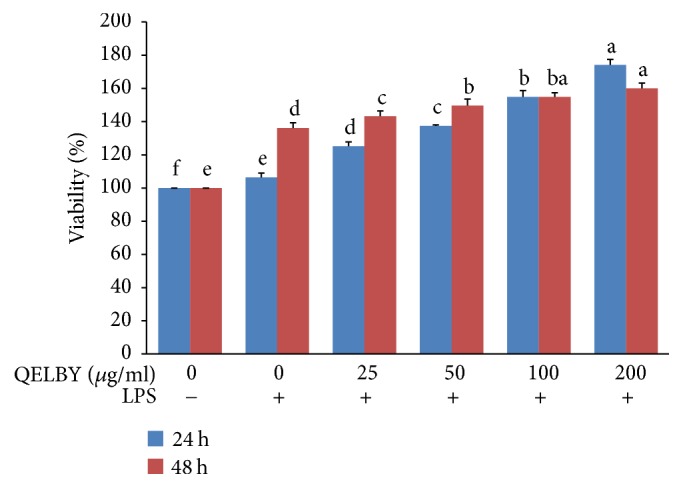
*Evaluation of the viability of RAW 264.7 macrophage cell.* Culture media were prepared by mixing with different amount of suspended layer from the DMEM mixed with 1% of QELBY ceramic powder and left for 48 hours. The blue and red bars indicate the length of incubating time, 24 hours and 48 hours, respectively, with the prepared media. Data are mean ± SD (*n* = 4). Bars with different superscript for each standing time are significantly different at *p* < 0.05.

The viability of RAW 264.7 macrophage cell increased in a dose dependent manner in the amount of the added suspended layer as shown in Figure 8. Compared to the control, it increased 1.5-fold in a media of 200 *μ*g/ml concentration. There was no significant difference in the viability of cells regardless of incubation time length (24 hours or 48 hours). Note that all of the samples incubated with the prepared culture media after LPS stimulation showed a higher viability compared to the samples treated with LPS stimulation only or incubated without LPS stimulation.

**Figure 9:**
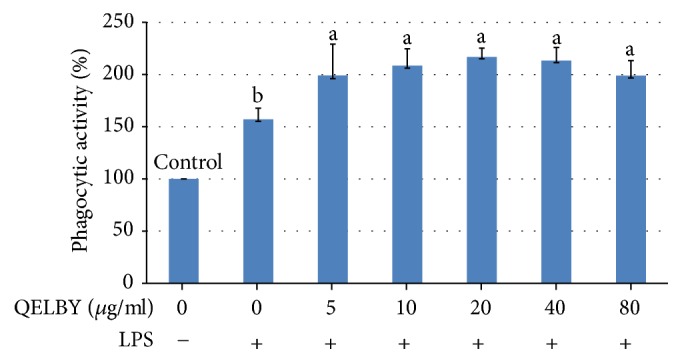
*Evaluation of phagocytic activity.* Phagocytic activity was measured by the uptake of neutral red dye. Culture media were prepared by mixing different amounts of suspended layers from the DMEM mixed with 1% of QELBY powder and left for 48 hours. Data are mean ± SD (*n* = 5). Bars with different superscript are significantly different at *p* < 0.05.

The prepared culture media increased the phagocytic activity also as shown in Figure 9. The activity almost doubled for the concentration of 5 *μ*g/ml and almost the same level of activity was maintained up to the 80 *μ*g/ml. Unlike the viability, the dose dependent manner was not observed. For reference, the activity of RAW 264.5 macrophage cell stimulated with LPS only increased about 1.5-fold compared to the control.

**Figure 10:**
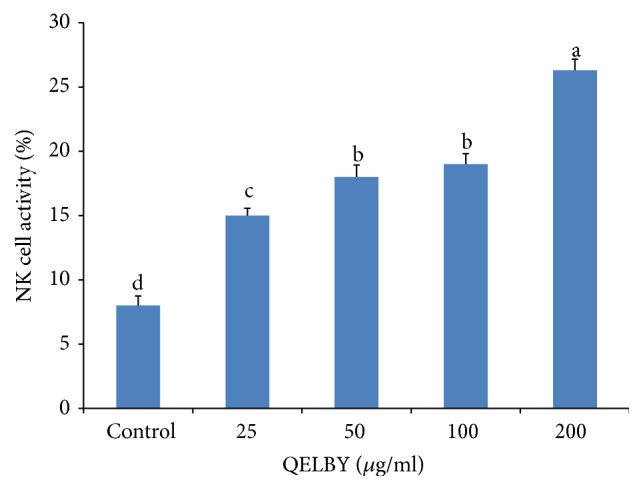
*Evaluation of natural killer cell activity.* Effector cell (isolated spleen cell) and target cells (YAC-I) were cultured at an effector : target ratio of 10 : 1 in RPMI 1640 media. The culturing media were prepared by mixing with different amount of suspended layer from the RPMI 1640 media mixed with 1% of QELBY powder and left for 24 hours. NK cell activity was assessed using CCK-8 assay to measure cell cytotoxicity against YAC-I cells. The results are expressed as mean ± SD (*n* = 5). Bars with different superscript are significantly different at *p* < 0.05.

A measurement of NK cell activity shows that the prepared media has a positive effect in a dose dependent manner as shown in Figure 10, resulting in a 3-fold increase for the medium mixed with 200 *μ*g/ml of suspended layer.

**Figure 11:**
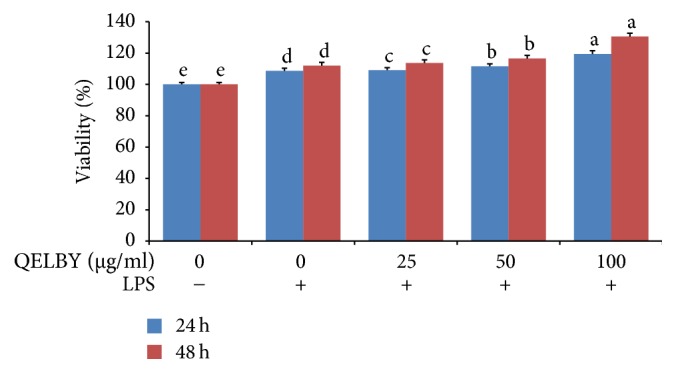
*Evaluation of viability of mouse splenocytes incubated for 24 and 48 hours.* The data shows a significant dose dependent effect on the viability of mouse splenocytes. The results are expressed as mean ± SD (*n* = 5). Bars with different superscript are significantly different at *p* < 0.05.

**Figure 12:**
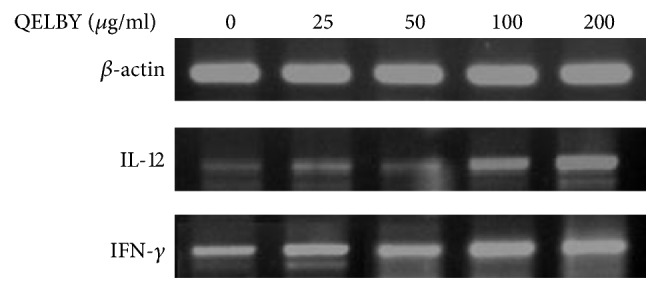
*Changes in cytokine expression of murine splenocytes incubated with the prepared media for 24 hours.* Reverse transcription polymerase chain reaction** (**RT-PCR) shows that both L-12 and INF-*γ* expressions were upregulated dose dependently by the incubation with the prepared media.

The incubation of mouse splenocytes with the prepared media showed a significant dose dependent effect on the viability as shown in Figure 11 although it is not as dramatic as in the case of phagocytic activity. The reverse transcription polymerase chain reaction (RT-PCR) shows that both interleukin-12 (IL-12) and interferon-gamma (INF-*γ*) expressions were upregulated dose dependently by incubating with the prepared media as shown in Figure 12. This result confirms the increased activity of the splenocyte cells shown in Figure 11.

#### 3.2.2. Culturing Media Prepared with Water Treated in the Noncontact Method

**Figure 13:**
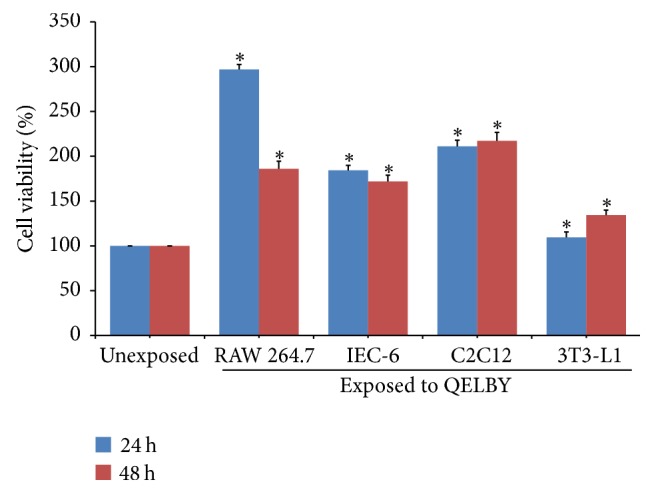
*The effects of the media prepared with the deionized water treated in a noncontact manner on the viability of various cells.* The deionized water was treated for 48 hours. Blue and red bars indicate the length of incubating time, 24 hours and 48 hours, respectively. Data are mean ± SD (*n* = 6). Bars with (*∗*) are significantly different compared to their unexposed counterpart group (*p* < 0.05).

For the culture medium prepared by mixing the DMEM powder with only deionized water treated in a noncontact manner, the viability of the macrophage cell was almost doubled or tripled depending on the length of incubation time as shown in Figure 13. Since the macrophage cell is sensitive to stimulation from outside as an immune cell, other kinds of normal cells besides macrophage cell, including Rattus norvegicus (IEC-6), mouse myoblast (C2C12), and murine adipocyte (3T3-L1), were incubated in the same way to confirm whether the same tendency is observed or not. Although there were some differences in the level of viability, all of them showed the same tendency, that is, increased cell viability as shown in Figure 13. The effect of incubation time was not so large compared to that of the macrophage cell. The unexposed deionized water was used for the preparation of control for all kinds of cells.

**Figure 14:**
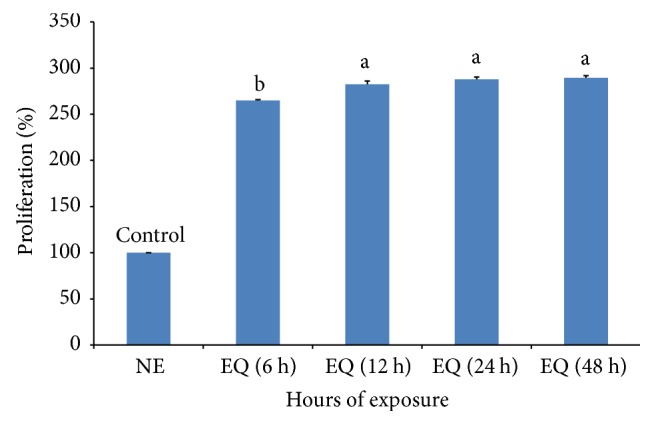
*The effects of the exposure time on the viability of RAW 264.7 macrophage cell.* Deionized water was exposed to the QELBY ceramic powder for a specified time at room temperature before preparation of DMEM. Cells were seeded in different plates for each exposure time (NE: DMEM was prepared with the water, which was not exposed; EQ: DMEM prepared with the water, which was exposed to QELBY powder for specified times). Data are mean ± SD (*n* = 7). Bars with different superscript are significantly different at *p* < 0.05.

To observe the effect of the difference in exposure time on the viability of macrophage cell, culture media was prepared using deionized water which was treated in the noncontact manner for 6, 12, 24, and 48 hours, respectively. All of them showed an almost 3-fold increase in viability with a slight difference among them as shown in Figure 14. This means that even a short period of exposure time shorter than 6 hours may be enough to increase the viability of macrophage cell.

#### 3.2.3. Effect of Culturing Media on a Breast Cancer Cell

**Figure 15:**
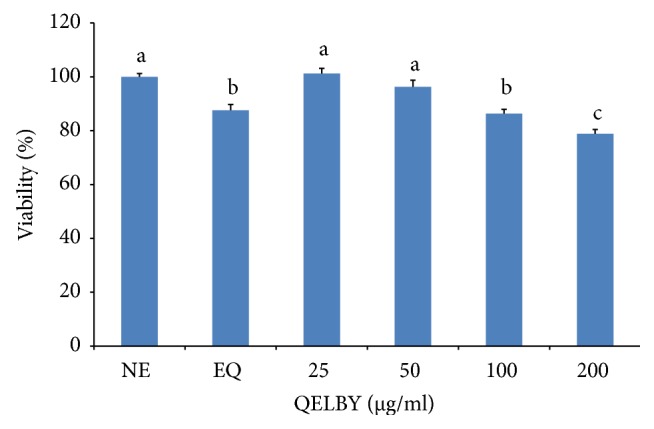
*The effect of the culturing media on the viability of a breast cancer cell, MCF-7.* NE in the diagram indicates that unexposed deionized water was used to prepare the medium as a control. EQ means that deionized water treated in the noncontact manner for 48 hours was used for the preparation of medium. The number indicates the amount of the suspended layer which was taken from the premixed DMEM with 1% of QELBY® ceramic powder and left for 48 hours to prepare the media. Data are mean ± SD (*n* = 6). Means with different superscript are significantly different at *p* < 0.05.

We performed the same experiment to observe its effect on the viability of an abnormal cell. A breast cancer cell, MCF-7, was incubated in the same manner and the viability was suppressed in a concentration-dependent manner as shown in Figure 15. It was inhibited to a level below 80% compared to the control for a concentration of 200 *μ*g/ml. The same trend was observed in the media prepared with a water treated in a noncontact manner for 48 hours as indicated by the bar of EQ in Figure 15.

**Figure 16:**
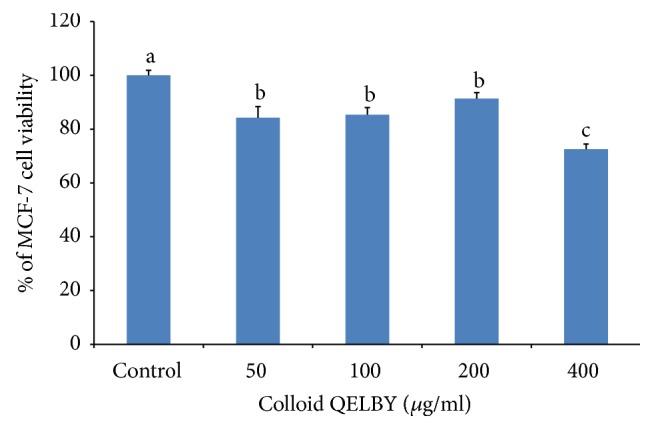
*The effect of the QELBY colloid on the viability of MCF-7 breast cancer cells.* The QELBY colloid was prepared by mixing with 1% of QELBY power and DMEM and centrifuged at 3,000 rpm. A different amount of the supernatant was used for the preparation of DMEM media. Data are mean ± SD (*n* = 4). Means with different superscript are significantly different at *p* < 0.05.

To see whether there is any influence on the cell viability of preparation process, the QELBY ceramic powder was mixed with the DMEM and centrifuged for 5 minutes right after mixing with the powder rather than waiting for 48 hours of standing time. The supernatant after the centrifuge was used for the preparation of culturing media in a different amount. Results shown in Figure 16 indicate that the effect is slightly lower for the same concentration, 100 and 200 *μ*g/ml each, compared to those of the data shown in Figure 15. At the concentration of 400 *μ*g/ml, the viability was suppressed almost to 70% of the control. This result implies that 48 hours of standing time may not be necessary to see the effect of the culturing media prepared in a contact method.

## 4. Discussions

There has been an argument regarding the physicochemical properties of interfacial water and the possibility that it may influence biological activities depending on the hydrophobic or hydrophilic properties of the cell membrane and other organic components of cells. It was claimed that water in contact with a hydrophobic surface will have a lower density and a better adsorption of various surfactants and proteins from water [14]. This approach is based only on the variation of the density of interfacial water.

It has been reported that the increase of the ultra-weak photon emission is associated with an oxidation reaction. The number of photon emissions is reduced when an antioxidant component such as catechin is added to water [15] while it increases under an oxidative atmosphere [16, 17]. Therefore, the number of photon emissions is expected to decrease for water with an antioxidant property. In this study, it was clearly demonstrated that the structured water prepared through a mixture with the hydrophilic ceramic powder exhibits an antioxidant property. Antioxidants inhibit the oxidation of lipids, proteins, or other molecular components in cells [18], and the generation of an ultra-weak photon emission is also associated with the oxidation of proteins and amino acids in bean sprout stems [19]. This indicates not only that there is a variation in the density of water, but also that an electric property can be a critical factor in the role of water. Actually, it has been found that an electric potential as high as −200 mV exists across the boundary between the exclusion zone and outside of this region (exclusion zone negative), in addition to the difference in density [6].

Interestingly, the water treated in the noncontact manner also demonstrates an antioxidant property as shown in Figures 6 and 7. The level of antioxidant property increases with longer exposure time. The energy source for the formation of structured water in the noncontact manner is possibly the infrared rays radiating from the ceramic powder [20–22]. This technique is quite important in the analysis of experimental results since only the structure of water was modified without any addition of material components to the water.

The modification of water structure seems to be a sort of continuous and gradual phenomenon. According to the experimental results of the ultra-weak photon emission (Figures 6 and 7) which was carried out for 93 hours in the noncontact manner, the water can be structured for quite a long time continuously through exposure to the powder for a longer time. However, the effects of exposure time on cell viability show that less than 6 hours is enough to obtain the increased viability of the RAW-264.7 cell (Figure 14). This implies that it may not be necessary to make a highly structured water by treating the water for a longer time for biological purposes. In other words, cells are very sensitive to the structure of water, which is supported by the fact that even a very small amount of premixed structured water, 0.5% (Figure 9), is effective in increasing phagocytic activity. Additionally, the structured water is stable enough to show these effects, even when mixed with a new fresh culture medium and shaken well. Only a small amount of structured water is necessary to increase bioactivities of cells as shown in Figure 8.

The culturing media mixed with QELBY powder or prepared with the antioxidant structured water exhibit an increase in the viability of normal cells. Recognizing that the same tendency was observed for both kinds of media prepared either by mixing with the QELBY powder or by mixing with the deionized water treated in the noncontact manner, the experimental results reported here must be interpreted as it relates to the antioxidant property of water generated by the modification of water structure, rather than the material components.

It is expected that a reactive oxygen species scavenging effect may occur through this media as other nutritional antioxidants [23–26]. As a result, the electron transport chain in the mitochondrion membrane may be activated, resulting in the increased viability for normal cells. Considering the principle of the CCK-8 assay kit, this explanation is more convincing since the formazan dye is formed as tetrazolium salt in the CCK-8 solution which is reduced by the dehydrogenase in the electron transport chain.

Another possibility is in the increased proton concentration. When the electric potential is generated between the exclusion zone and outside of it, a large amount of protons is generated in the outside region [6]. This means that the supply of proton can be enhanced when the water is structured. Considering the fact that the mitochondrial membrane potential, Ψm, is composed of potentials from ion and proton gradients, the increased amount of proton may contribute to the increased Ψm. Actually, it was reported that Ψm was increased up to 1.36-fold for RAW 264.7 and HD11 cells after exposing them to the QELBY powder in the noncontact manner for 48 or 72 hours [27]. The increased Ψm may contribute to the increased viability and increased ATP production. However, this was not observed for these cells.

On the other hand, the same culturing media show the opposite trend for malignant cancer cells. This is quite an interesting phenomenon since the same stimulator results in the opposite reactions depend on the cells, normal or malignant. At this time, the mechanism is unclear, but the increased Ψm may provide clues for further research. According to the previous report, the Ψm was observed to increase also for the HeLa cell by more than 1.36-fold, while the intracellular ATP level decreased after 72 hours of exposure [27]. It is well known that cancer cells have a more hyperpolarised Ψm than normal cells [28–32] and can be larger than 50% than normal cells [33]. It has been reported that 10% value alterations from its optimum in Ψm can cause serious variability on ATP synthesis, by as large as a 90% decrease [34]. Thus, the hyperpolarity in Ψm of a cancer cell may be related to the malfunction of the electron transport chain, that is, oxidative phosphorylation. Warburg reported that cancer cells' metabolism occurs through aerobic glycolysis unlike normal cells, which rely on aerobic respiration [35]. The increase in Ψm and decrease in ATP production in the HeLa cell cultured in the noncontact manner with the QELBY powder imply that the variation in Ψm is one of the causes for the decreased viability of the cancer cell, MCF-7. Definitely, experiments on the effect of structured water on the potential Ψm is required for a detailed mechanism.

It has been claimed that continued oxidative stress can lead to chronic inflammation, which in turn could mediate most chronic diseases including cancer [36, 37]. In this report, the antioxidant property of the structured water, increased viability and cytokine expression, and suppression of cancer cell were described. More detailed anti-inflammatory effects of QELBY powder were reported elsewhere [38]. It is presumed that all of these results, antioxidant, anti-inflammation, and anticancer effects of QELBY ceramic powder, are strongly correlated to each other. Additionally, it was observed that DNA exposed to QELBY was protected from the damage caused by free radicals [27]. These results suggest that a future study on the effect of QELBY should focus on its overall holistic influence on a living body rather than focusing on molecular level cell cycle regulatory system.

Again, these results strongly suggest that water plays a critical role in cellular metabolism, more than previously understood. Water is not merely a simple liquid, but instead plays an important role in cellular and molecular mechanisms by stimulating the enhanced bioactivities of normal cells, while suppressing those of malignant cancer cells. All of these results strongly point to the positive role of structured water in cellular bioactivities.

## 5. Conclusion

The experimental results reported here imply that both the materialistic nutritional components of culture media and water structure can be critical factors in the analysis of bioactivities of cells. Structured water, either prepared through mixture with a hydrophilic ceramic powder, or treated in the noncontact manner, exhibited antioxidant property. The increase in cellular bioactivities is strongly related to the antioxidant property of structured water. Structured water appears to exhibit the same properties as an antioxidant. Since no antioxidant components were added in the water treated in the noncontact method, these properties are solely related to the structure of water. These experimental results strongly suggest that water can play an essential role in bioactivities of cells, depending on its antioxidant effects, based on its structure. Water itself can be an active constituent in cell biology, as are many other cellular molecules.

## Supplementary Material

Schematic diagrams showing the overall preparation process of media and cell viability measurement procedure.

## Figures and Tables

**Table 1:** *Average number of ultra-weak photon emissions per one second*. Measurements were taken for eight minutes, from bean sprout stems stored in deionized water only, water mixed with the silica powder, and water mixed with the QELBY ceramic powder.

Time of measurement	16 hours later	6 days later	7 days later
(1) Control, deionized water only	27.86	27.97	35.65
(2) Deionized water + silica (0.1 g/ml)	30.31	39.49	39.20
(3) Deionized water + QELBY (0.1 g/ml)	27.28	18.16	23.78
