# Supplementary material for: Effect of Antioxidant Water on the Bioactivities of Cells
Source: Int J Cell Biol. 2017 Aug 17;2017:1917239. doi: 10.1155/2017/1917239 (PMC5585622; doi:10.1155/2017/1917239)
Supplement: Supplementary file 1 — Schematic diagrams showing the overall preparation process of media and cell viability measurement procedure. [file 1917239.f1.docx]

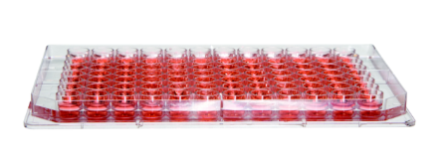


**Media prepared with pre-mixed DMEM**


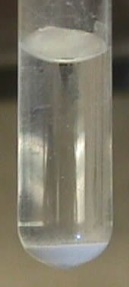

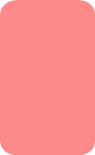


**QELBY powder powder**

**Cells were seeded in a 96 well plate at a density of 1x10_5_ cells/well.**

**Allowed to adhere for 3 hours.**

**Treated with LPS 10 ㎍/㎖**

**(lipopolysaccharid)**

**30 ㎖ of DMEM + 0.3 g of QELBY powder**

**Allowed to stand for 2 days at 4 ℃ after strong agitation for 5 min.**

**.**

**Suspended layer was added in different amount to each well,**

**0, 50, 100, 200 μg/ml**

**Incubated at 37 °C and 5 % CO_2_ for 24 hours.**

**10 µl of CCK-8 solution were added to each well and incubated further for 2 hours.**

**Absorbance was measured by ELISA reader at 450 nm wavelength**

**SFig 1. Block diagram showing the cell culture process using a medium prepared by mixing with the suspended layer from the pre-mixed DMEM with the QELBY powder for the measurement of cell viability.**

**Absorbance was measured by ELISA reader at 450 nm wavelength**


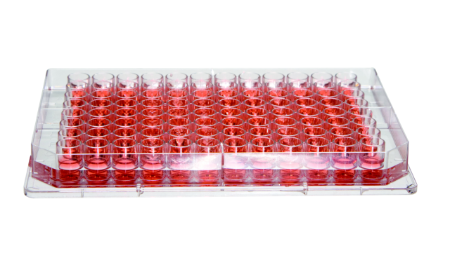


**Phagocytic activity measurement**


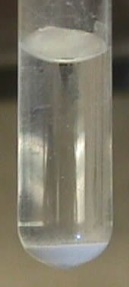

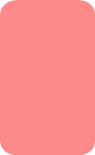


**QELBY powder**

**Cells were seeded in a 96 well plate at a density of 1x10_5_ cells/well.**

**Allowed to adhere for 3 hours.**

**Treated with LPS 10 ㎍/㎖**

**(lipopolysaccharid)**

**10㎖ of DMEM+**

**0.1g of QELBY powder**

**Allowd to stand for 2 days after strong**

**agitation (5 min).**

**Different amount of suspended layer was added to each well,**

**0, 50, 100, 200 μg/ml**

**Absorbance was measured by ELISA reader at 550 nm wavelength**

**Incubated at 37 °C in humidified 5 % CO_2_ for 24 hours.**

**100 µl of 0.075 % aseptic neutral red solution was added and incubated further for 1 hour.**

**Plate was washed 3 times with PBS**

**and a mixture of 100 % ethanol and 99.9 % acetic acid (1:1 v/v) was added to 150 ㎕ of cell lysate.**

**Absorbance was measured by ELISA reader at 550 nm wavelength**

**SFig. 2 Block diagram showing the cell culture process using a medium prepared by mixing with the suspended layer from the pre-mixed DMEM with the QELBY powder for the measurement of phagocytic activity.**

**Natural Killer cell activity measurement**

**Target cell (YAC-1) was seeded in a 96 well plate at a density of 5x10_3_cells/well and allowed to adhere for 3 hrs.**

**Target cell and Effector cell were mixed at a ratio (v/o) of;**

**Effector : Target = 10 : 1**

**Effector cell was prepared by isolation of spleen cell from a mouse.**

**Different amount of suspended layer was added to fresh RPMI 1640 media, 0, 50, 100, 200 μg/㎖**

**10 ㎖ of RPMI 1640 + 0.1 g of QELBY powder**

**Allowed to stand for 2 days after strong agitation (5 min).**


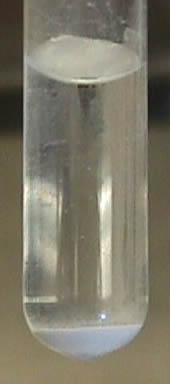

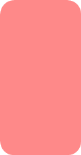


**Qelby powder**

**Incubated in RPMI 1640 media for 24 hours.**

**CCK-8 assay for cytotoxicity measurement against YAC-1 cell.**

**Absorbance was measured by ELISA reader at 450 nm wavelength**

**SFig. 3. Block diagram showing the cell culture process using a medium prepared by mixing with the suspended layer from the pre-mixed RPMI 1640 with the QELBY powder for the measurement of natural killer cell activity.**

**Media prepared with non-contact treated water**

***Dulbeco’s Modified Eagle’s Media (powder)***

**50 mℓ of DW in a conical tube was put in QELBY powder for 2 days.**

**Exposed water was used for the preparation of culture media by mixing with DMEM**

**Cells were seeded in a 96 well plate at a density of 1x10_5_cells/well.**

**Allowed to adhere for 3 hours.**


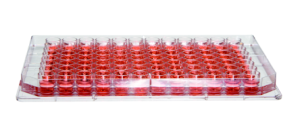


**Treated with LPS 10 ㎍/㎖ (lipopolysaccharid)**

**Incubated at 37 °C with 5 % CO_2_ for 24 hours.**

**10 µl of CCK-8 solution was added to each well and incubated further for 2 hours.**

**Absorbance was measured by ELISA reader at 450 nm wavelength**

**SFig 4. Block diagram showing the cell culture process using a medium prepared with the deionized water treated in a non-contact manner for the measurement of cell viability.**

**Absorbance was measured by ELISA reader at 450 nm wavelength**
